# GeoSieveNet: An Image Classification Network with Geometry-Adaptive Sampling and Hierarchical Feature Sifting

**DOI:** 10.3390/jimaging12100504

**Published:** 2026-10-12

**Authors:** Haixu Chen, Zhen Wang, Yang Liu, Mingzhe Yang, Gaoquan Ming, Lujia Yang

**Affiliations:** 1 School of Computer Science and Technology, Shandong University of Technology, Zibo 255000, China; 22805050004@stumail.sdut.edu.cn (H.C.); 25505020700@stumail.sdut.edu.cn (Y.L.); 25505040751@stumail.sdut.edu.cn (M.Y.); 22110507052@stumail.sdut.edu.cn (G.M.); 2 Mathematics and Applied Mathematics (Data Analysis), Wenzhou-Kean University, Wenzhou 325000, China; yangluj@kean.edu

**Keywords:** image classification, residual network, geometry-adaptive sampling, rotational deformable convolution, hierarchical gated attention

## Abstract

Standard convolution samples features on a fixed grid whose locations do not adapt to the input content. We develop the Geometry-Adaptive Sampling and Hierarchical Feature-Sifting Network (GeoSieveNet) based on a 34-layer residual network (ResNet-34). The rotational deformable convolution module (RDCM) assigns predefined offsets with different orientations to individual channel groups and combines them with input-predicted dynamic offsets to adjust sampling locations. Its feature preservation module (FPM) fuses responses from the fixed-grid and rotational deformable branches. Hierarchical gated attention (HGA) combines directional statistics with local convolutional responses to generate spatial gating weights within each channel group. GeoSieveNet achieved classification accuracies of 96.95%, 81.86%, 94.71%, and 89.79% on CIFAR-10, CIFAR-100, Imagenette, and Imagewoof, respectively. Across three independent runs, these results exceeded ResNet-34 under the same training settings by 0.66, 3.06, 4.73, and 5.19 percentage points, respectively. All paired tests were significant at the 0.001 level. Ablation experiments attributed gains of 0.36–1.89 percentage points to FPM alone, with larger gains on datasets exhibiting greater geometric variation. At the same floating-point operation budget as ResNet-34, GeoSieveNet retained an accuracy advantage of 0.54–3.96 percentage points.

## 1. Introduction

Changes in object pose and viewing direction alter the locations of edges, textures, and local parts in feature maps. AlexNet, VGG, the 34-layer residual network (ResNet-34), DenseNet, and EfficientNet differ in architecture and scale, but their main convolutions still sample on regular grids [1,2,3,4,5]. Data augmentation introduces variations in orientation and scale into the training set, while the sampling procedure of the convolution operator remains unchanged.

Group-equivariant convolutional neural networks (G-CNNs) and harmonic networks constrain feature transformations through symmetry [6,7]. Deformable convolutional networks (DCNs) learn sampling offsets from the input, allowing the sampling region to adapt to image content [8,9]. These approaches address geometric variation through transformation constraints and sampling locations, respectively. They motivate combining predefined orientations with input-dependent offsets in convolution.

Sampling coordinates determine where convolution reads features, whereas attention within residual blocks adjusts response magnitudes. Squeeze-and-excitation (SE) modules generate weights from channel statistics [10]. The convolutional block attention module (CBAM) computes channel and spatial weights [11], while coordinate attention preserves positional information along two spatial directions [12].

We propose the Geometry-Adaptive Sampling and Hierarchical Feature-Sifting Network (GeoSieveNet), which combines direction-dependent dynamic sampling with within-group feature weighting in ResNet-34. The rotational deformable convolution module (RDCM) adjusts sampling locations, while hierarchical gated attention (HGA) generates feature weights from directional statistics and local responses.

The main contributions are as follows:

1. We propose RDCM, which assigns direction-dependent predefined offsets to channel groups and combines them with input-predicted dynamic offsets to adjust sampling locations. Its feature preservation module (FPM) fuses responses from the fixed-grid and rotational deformable branches. Independent ablations attribute gains of 0.36–1.89 percentage points to FPM, with larger gains on datasets exhibiting greater geometric variation.
2. We design HGA to combine horizontal and vertical statistics with local convolutional responses within channel groups. The resulting spatial gating weights reweight the residual features.
3. We construct GeoSieveNet by integrating RDCM and HGA into ResNet-34. On four primary classification datasets, we report means and standard deviations across 3 independent runs, paired significance tests, comparisons at matched floating-point operation and parameter budgets, module ablations, and computational cost and inference-speed measurements. Accuracy is significantly higher than that of ResNet-34 under the same training settings on all four datasets, and the advantage persists at the same floating-point operation budget.

## 2. Related Work

### 2.1. Geometry-Adaptive Convolution and Deformable Sampling

Geometric adaptation extends standard convolution primarily through transformation constraints and sampling locations. G-CNNs generalize convolution to groups comprising translations, discrete rotations, and reflections. Weight sharing across transformations preserves the correspondence between input and feature transformations [6]. Harmonic networks use circular harmonic filters to relate rotation angles to the phase of feature responses, yielding representations equivariant to continuous rotations [7]. These approaches incorporate selected geometric transformations into the architecture, with response behavior constrained by the transformation group or filter formulation.

Deformable convolution adapts to local geometric variation by changing sampling locations. The first generation of deformable convolutional networks (DCNv1) augments a regular grid with two-dimensional offsets predicted from the input features. This allows a single convolutional layer to use different sampling patterns at different locations [8]. Deformable ConvNets V2 (DCNv2) further introduces a modulation coefficient for each sampling point, jointly adjusting its location and contribution to the output. It also expands the use of deformable convolution in the backbone [9]. Both versions learn offsets through the target task without prescribing the transformation type for each local region.

InternImage uses the third generation of deformable convolution (DCNv3) as its core operator, extending input-dependent spatial aggregation to large-scale vision backbones [13]. DCNv3 uses group-wise aggregation, with each group learning its own sampling offsets and modulation weights, which are normalized over sampling points. Projection weights are shared across sampling points within each group, so projection parameters perform channel mapping while sampling weights govern spatial aggregation. Standard convolution assigns separate projection weights to different kernel locations. The fourth generation of deformable convolution (DCNv4) simplifies spatial aggregation and improves operator efficiency. It shows that shared within-group projections and grouped sampling can jointly support accuracy and speed in large-scale backbones [14]. Geometry-adaptive methods differ in whether transformation relationships are prescribed and how location adjustment and response weighting jointly contribute to aggregation. Rotation-equivariant representations emphasize explicit transformation correspondences, whereas deformable sampling emphasizes input-dependent local sampling locations. These approaches provide distinct ways to model geometry. RDCM retains grouped sampling and shared within-group projections, but explicitly assigns a predefined orientation offset to each channel group; dynamic offsets refine this displacement. Beyond geometric adaptation, recent classification methods have explored attention architectures, frequency-domain enhancement, and robustness distillation. Couplformer reorganizes coupled attention in vision Transformers [15], while MMA-CCT strengthens global modeling through multi-stream hybrid attention [16]. ViT-Lite investigates pretraining lightweight Transformers on small datasets [17]. Fast multi-attention dynamic convolution (FMDConv) balances speed and accuracy [18], and cyclic iterative adversarial robustness distillation (CIARD) improves representation quality [19]. DenseNet with channel-wise and feature-point reweighting (CAPR-DenseNet) adjusts the weights of channels and feature points [20]. These methods focus on weight computation or feature transformation while retaining fixed-grid sampling, providing a comparison with our joint adjustment of sampling locations and response weights.

### 2.2. Attention Mechanisms and Feature Reweighting

Attention mechanisms use input-dependent weights to select and aggregate features. Self-attention computes weights from query–key similarities and forms weighted combinations of values, establishing dependencies across positions [21]. In convolutional networks, SE modules aggregate spatial information into channel descriptors and use them to generate channel weights [10]. Global average pooling summarizes each channel by its mean response over the feature map, discarding the spatial origin of individual responses. Self-attention directly organizes interactions between positions, whereas SE primarily adjusts relative channel response magnitudes. All positions within a channel share the same scaling coefficient.

CBAM sequentially computes channel and spatial attention, distinguishing responses across spatial locations in addition to selecting channels [11]. Its channel weights are shared across spatial locations, while its spatial weights are shared across channels. To generate channel weights, CBAM applies spatial average and max pooling to summarize mean and strongest responses, respectively. A shared multilayer perceptron processes both descriptors. To generate spatial weights, it computes the same statistics along the channel dimension and applies convolution to produce position-specific weights. The two branches use the same statistical operations but preserve information along different dimensions. Coordinate attention decomposes two-dimensional global pooling into one-dimensional encodings along the two spatial directions. Aggregation along one direction preserves positional indices along the other, producing direction-aware and position-sensitive weights [12]. These designs reflect different trade-offs among global channel statistics, spatial responses, and directional positional encoding.

Reweighting modules primarily use spatial information to estimate response magnitudes. Even when weights vary across positions, features retain their original coordinates, whereas deformable convolution directly shifts sampling points. DCNv2 modulation also applies weighting, but its weights act on sampling points during convolutional aggregation. SE, CBAM, and coordinate attention instead generate channel or positional weights for existing features. This study combines dynamic sampling with predefined orientations and within-group feature reweighting for image classification.

## 3. Methods

### 3.1. Problem Formulation and GeoSieveNet Architecture

**Figure 1:**
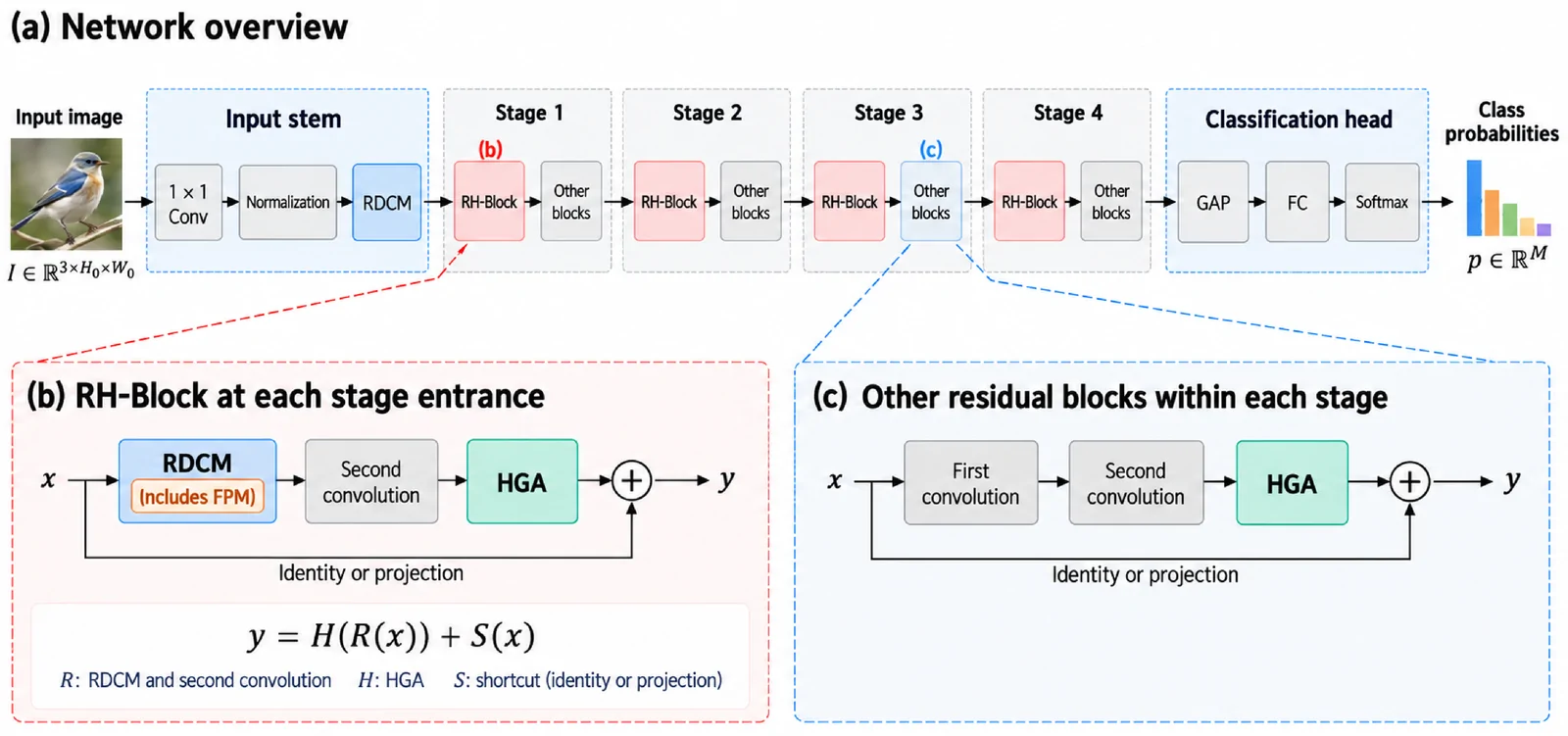
GeoSieveNet architecture and residual blocks. (**a**) Input, four-stage residual structure and classification head. GAP and FC denote global average pooling and the fully connected layer, respectively. (**b**) Rotational hierarchical block (RH-Block) at the beginning of each stage. The rotational deformable convolution module (RDCM) contains the feature preservation module (FPM). Hierarchical gated attention (HGA) is placed after the second convolution and before residual addition. (**c**) The remaining residual blocks within each stage retain conventional convolutions, with HGA in the same position.

Let the input image be $I\in R^{3\times H_{0}\times W_{0}}$ and the number of classes be *M*. The classification network is denoted by f(I;Θ), with a fully connected layer and softmax producing class probabilities. GeoSieveNet retains the four-stage residual structure of ResNet-34, as shown in Figure 1a. RDCM is used at the input and in the first residual block of each stage. Each stage begins with a rotational hierarchical block (RH-Block), which replaces the first conventional convolution with RDCM, as shown in Figure 1b. The remaining residual blocks within each stage retain two conventional convolutions, as shown in Figure 1c. Both block types place HGA after the second convolution and before residual addition. The shortcut branch uses either an identity mapping or a projection mapping.

A 1×1 convolution adjusts the input image channels, followed by batch normalization (BN) before RDCM. Global average pooling (GAP) compresses the final-stage features, and a fully connected (FC) layer maps them to classes. Softmax converts the outputs into class probabilities.

### 3.2. Rotational Deformable Convolution Module

**Figure 2:**
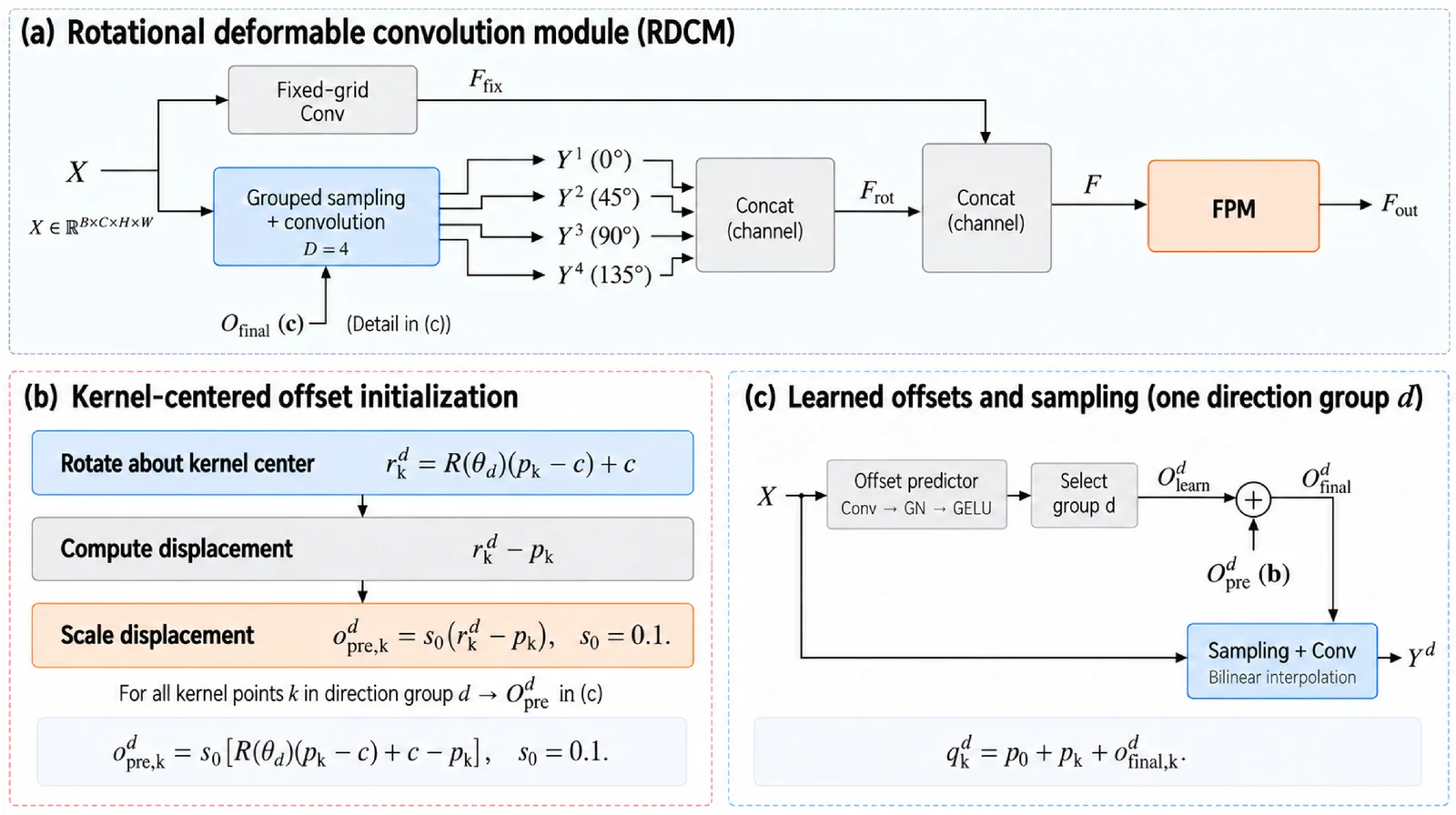
RDCM architecture and sampling procedure. (**a**) Response concatenation and FPM fusion for the fixed-grid branch and the rotational deformable branch with four direction groups. (**b**) Rotational offset initialization about the convolutional kernel center and offset scaling, with $s_{0}=0.1$. (**c**) Learned offsets, final sampling offsets and convolution with bilinear sampling for direction group *d*. GN and GELU denote group normalization and the Gaussian error linear unit, respectively.

RDCM samples the input in parallel through a fixed-grid branch and a rotational deformable branch, as shown in Figure 2a. The fixed-grid branch samples at regular grid locations. The deformable branch partitions the output channels into *D* groups, each assigned an initial direction. Dynamic offsets predicted from the input features further adjust the sampling locations. The responses of all direction groups are first concatenated along the channel dimension, yielding the deformable branch output $F_{\mathrm{rot}}=\mathrm{Concat}\left(Y^{1},\ldots ,Y^{D}\right)$. This output is then concatenated with the fixed-grid branch output $F_{\mathrm{fix}}$ along the channel dimension. The resulting features $F=\mathrm{Concat}(F_{\mathrm{fix}},F_{\mathrm{rot}})$ are passed to FPM.

For a K×K convolutional kernel, let $p_{k}$ denote the *k*th regular-grid coordinate and c the geometric center. Direction group *d* has an initial angle $\theta_{d}$ and an initial offset scale $s_{0}$. The predefined rotational offset is

(1) $$o_{\mathrm{pre},k}^{d}=s_{0}\left[R\left(\theta_{d}\right)\left(p_{k}-c\right)+c-p_{k}\right],$$

where $R(\theta_{d})$ is the two-dimensional rotation matrix. The grid coordinate after rotation about the kernel center is $r_{k}^{d}=R\left(\theta_{d}\right)\left(p_{k}-c\right)+c$. Scaling the displacement $r_{k}^{d}-p_{k}$ by $s_{0}$ gives the predefined offset, as illustrated in Figure 2b. Configuration experiments compare angle sets with two, four, and eight direction groups. The model uses four directions: 0°, 45°, 90°, and 135°.

Dynamic offsets are generated from the input features $X\in R^{B\times C\times H\times W}$. For offset features with spatial dimensions $H^{\prime }\times W^{\prime }$, the offset-prediction convolution produces $2DK^{2}$ channels. The raw offsets $O_{\mathrm{raw}}\in R^{B\times 2DK^{2}\times H^{\prime }\times W^{\prime }}$ are written as

(2) $$O_{\mathrm{raw}}=W_{o}*X+b_{o}.$$

The raw offsets are processed by group normalization (GN) and a Gaussian error linear unit (GELU) to obtain the learned offsets

(3) $$O_{\mathrm{learn}}=\mathrm{GELU}\left(\mathrm{GN}(O_{\mathrm{raw}})\right).$$

We partition $O_{\mathrm{learn}}$ into *D* groups along the channel dimension, with $K^{2}$ two-dimensional offsets per group. The learned offsets for direction group *d*, denoted by $O_{\mathrm{learn}}^{d}$, are added to the corresponding predefined rotational offsets to form the final sampling offsets

(4) $$O_{\mathrm{final}}^{d}=O_{\mathrm{pre}}^{d}+O_{\mathrm{learn}}^{d}.$$

Figure 2c illustrates offset generation and sampling for direction group *d*. The predefined rotational offsets and learned offsets jointly determine the sampling locations.

For output location $p_{0}$, the *k*th sampling location in direction group *d* is

(5) $$q_{k}^{d}=p_{0}+p_{k}+o_{\mathrm{final},k}^{d}.$$

Features at non-integer locations are read using bilinear interpolation. Let $g_{00}$, $g_{01}$, $g_{10}$, and $g_{11}$ be the four neighboring integer grid points of $q=(q_{x},q_{y})$. Their interpolation weights are

(6) $$\begin{array}{cccc} w_{00} & =(1-\alpha )(1-\beta ),\; & w_{01} & =(1-\alpha )\beta ,\\ w_{10} & =\alpha (1-\beta ),\; & w_{11} & =\alpha \beta , \end{array}$$

where $\alpha =q_{x}-\left\lfloor q_{x}\right\rfloor$ and $\beta =q_{y}-\left\lfloor q_{y}\right\rfloor$ are the fractional parts. The interpolated value is

(7) $$X\left(q\right)=\sum_{i\in \{0,1\}}\sum_{j\in \{0,1\}}w_{ij}\;X\left(g_{ij}\right),$$

Grid points outside the feature map are assigned zero values. The output of each direction group is

(8) $$Y^{d}\left(p_{0}\right)=\sum_{k=1}^{K^{2}}W_{k}^{d}\;X\left(q_{k}^{d}\right).$$

The convolutional responses of all direction groups are concatenated along the channel dimension to form $F_{\mathrm{rot}}$, corresponding to the first concatenation node in Figure 2a.

The implementation details of RDCM are as follows. Offset prediction uses a 3×3 depthwise separable convolution with padding 1 and stride 1. The offset features therefore retain the input spatial dimensions, $H^{\prime }=H$ and $W^{\prime }=W$. A 3×3 convolution with stride 2 precedes the input-stage RDCM, where $H^{\prime }=W^{\prime }=H_{0}/2=16$. The offset-prediction convolution produces $2DK^{2}=2\times 4\times 9=72$ channels in our configuration. These channels are divided equally into *D* groups, each containing $2K^{2}=18$ channels. Within each group, the channels are arranged as $K^{2}=9$ two-dimensional offsets in sampling-point order. Each pair follows the order (Δx,Δy), corresponding to channels 2k−1 and 2k in Equation (3). Projection weights are not shared across the *D* direction groups: group *d* uses its own $W_{k}^{d}$. The offset-prediction convolution parameters are shared throughout the network. Non-integer sampling locations $q_{k}^{d}$ are evaluated by bilinear interpolation with zero padding outside the feature map. A custom automatic differentiation function implements the forward and backward passes. Table 1 summarizes these settings.

### 3.3. Feature Preservation Within RDCM and Input-Stage Feature Extraction

**Figure 3:**
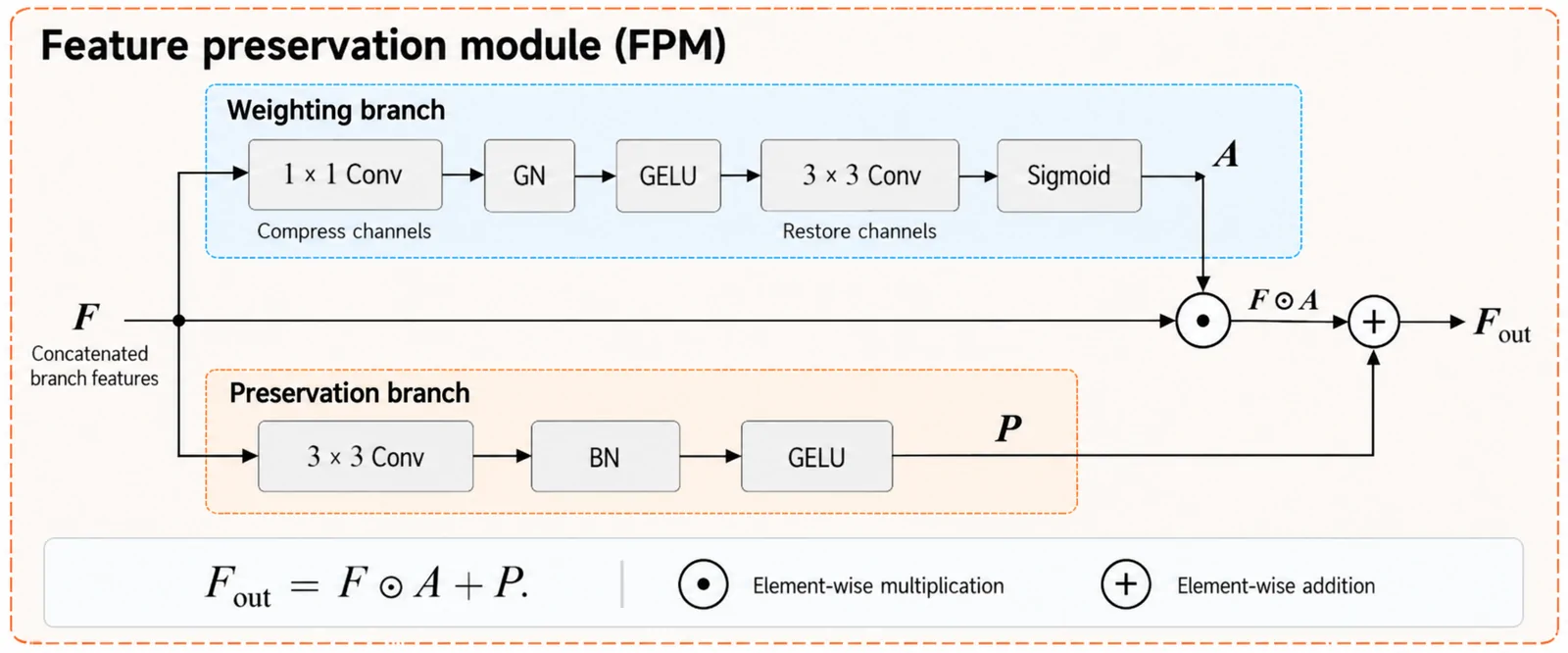
FPM architecture. The input F contains the concatenated features from the two branches. The weight branch produces A through a 1×1 convolution, GN, GELU, a 3×3 convolution, and Sigmoid. The preservation branch produces P through a 3×3 convolution, BN, and GELU. The output is $F_{\mathrm{out}}=F⊙A+P$.

RDCM uses FPM to process the concatenated features from its two branches. Figure 3 shows the weight and preservation branches of FPM. Let its input be $F\in R^{B\times C_{F}\times H\times W}$. In the weight branch, a 1×1 convolution reduces the channel count, followed by GN and GELU. A 3×3 convolution restores the channels, and Sigmoid produces the weights

(9) $$A=\mathrm{Sigmoid}\left(W_{2}*\mathrm{GELU}\left(\mathrm{GN}(W_{1}*F+b_{1})\right)+b_{2}\right).$$

The weights A have the same dimensions as F. The preservation branch applies a 3×3 convolution, BN, and GELU:

(10) $$P=\mathrm{GELU}\left(\mathrm{BN}(W_{3}*F+b_{3})\right).$$

The output of FPM is

(11) $$F_{\mathrm{out}}=F⊙A+P.$$

Equation (11) adds the convolutional branch output to the gated concatenated features. $F_{\mathrm{out}}$ retains the spatial dimensions of F and serves as the output of RDCM.

### 3.4. Hierarchical Gated Attention and the Rotational Hierarchical Block

**Figure 4:**
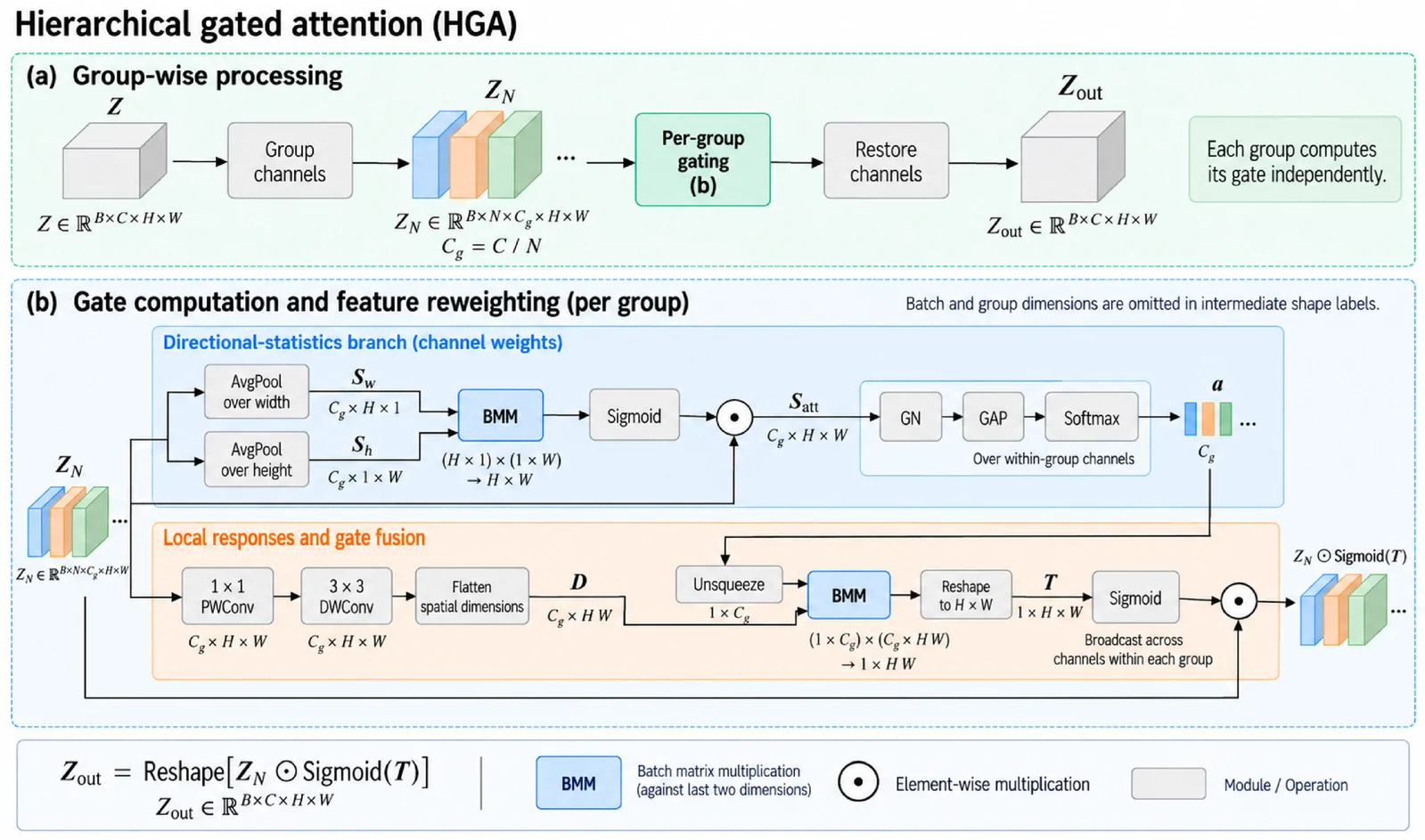
HGA architecture. (**a**) Channel grouping, independent gating within each group, and restoration of the original channel order. (**b**) Directional statistics produce within-group channel weights. Batch matrix multiplication (BMM) combines these weights with local convolutional responses to form spatial gates, which reweight the original grouped features. PWConv and DWConv denote pointwise convolution and depthwise convolution, respectively.

HGA receives features $Z\in R^{B\times C\times H\times W}$ and partitions the channels into *N* groups, each containing $C_{g}=C/N$ channels. The reshaped features are denoted by $Z_{N}\in R^{B\times N\times C_{g}\times H\times W}$. Gating is computed independently for each group before the original channel order is restored, as shown in Figure 4a. Figure 4b shows how directional statistics and local convolutional responses contribute to spatial gating.

Directional pooling retains statistics along a single spatial axis, as shown in the directional-statistics branch of Figure 4b. Averaging over width and height while retaining the pooled dimensions gives $S_{w}\in R^{B\times N\times C_{g}\times H\times 1}$ and $S_{h}\in R^{B\times N\times C_{g}\times 1\times W}$:

(12) $$S_{w}={\mathrm{AvgPool}}_{W}\left(Z_{N}\right),\;S_{h}={\mathrm{AvgPool}}_{H}\left(Z_{N}\right).$$

The first BMM combines the two statistics along their last two dimensions, forming two-dimensional statistics for each channel in each group. After Sigmoid, these statistics are multiplied elementwise by the original grouped features $Z_{N}$:

(13) $$S_{\mathrm{att}}=Z_{N}⊙\mathrm{Sigmoid}\left(\mathrm{BMM}(S_{w},S_{h})\right).$$

GN is applied to $S_{\mathrm{att}}$, and GAP compresses its spatial dimensions. Softmax normalizes the channel dimension within each group, producing within-group channel weights $a\in R^{B\times N\times C_{g}}$:

(14) $$a=\mathrm{Softmax}\left(\mathrm{GAP}(\mathrm{GN}\left(S_{\mathrm{att}}\right))\right).$$

The local path extracts spatial responses, corresponding to the local convolutional branch in Figure 4b. A 1×1 PWConv mixes channels within each group, and a 3×3 DWConv extracts information from spatial neighborhoods:

(15) $$D=\mathrm{Vec}\left({\mathrm{DWConv}}_{3\times 3}\left({\mathrm{PWConv}}_{1\times 1}\left(Z_{N}\right)\right)\right).$$

Flattening the spatial dimensions gives $D\in R^{B\times N\times C_{g}\times HW}$. The within-group channel weights a are expanded to $B\times N\times 1\times C_{g}$ and combined with the local responses through a second BMM. Reshaping yields an H×W gating map for each group:

(16) $$T={\mathrm{Reshape}}_{H\times W}\left(\mathrm{BMM}(\mathrm{unsqueeze}(a),D)\right),$$

Sigmoid converts the gating map $T\in R^{B\times N\times 1\times H\times W}$ into spatial weights, which are broadcast across channels within each group. These weights are multiplied elementwise by the original grouped features $Z_{N}$, and the original channel order is restored:

(17) $$Z_{\mathrm{out}}=\mathrm{Reshape}\left(Z_{N}⊙\mathrm{Sigmoid}\left(T\right)\right).$$

Each stage begins with an RH-Block, which replaces the first convolution with RDCM and places HGA after the second convolution. The other residual blocks retain their conventional first convolution and use HGA at the same position. Let R denote the mapping formed by RDCM and the second convolution, and let H denote HGA. The residual output of the RH-Block is

(18) y=H(R(x))+S(x),

where S is an identity mapping or a projection mapping for matching dimensions. Figure 1b shows the main path and shortcut connection of the RH-Block, while Figure 1c shows the other residual blocks within each stage.

## 4. Experiments and Results

### 4.1. Datasets and Experimental Scope

We used six datasets: CIFAR-10, CIFAR-100, Flowers-102, STL-10, Imagenette, and Imagewoof. Each was obtained in its default version from the official distribution channel, without additional version selection or custom splitting. Table 2 lists the input dimensions, class counts, split sizes, and actual usage in this study. Table 3 identifies the experiments performed on each dataset. CIFAR-10 and CIFAR-100 use the official training and test sets. Flowers-102 uses the official training, validation, and test sets, containing 1020, 1020, and 6149 images, respectively; all compared models use the same splits. STL-10 uses 5000 labeled training images and 8000 test images; its 100,000 unlabeled images are not used. Imagenette and Imagewoof use the training and validation sets provided by fastai, with the validation sets serving as test sets in this study. Training uses only the training sets, and test sets are excluded from model selection.

### 4.2. Training Configuration

Experiments ran on Ubuntu 22.04 with a single NVIDIA RTX 3090 graphics processing unit (GPU) with 24 GB of memory. The software environment comprised PyTorch 2.1.0, the Compute Unified Device Architecture (CUDA) 12.1, and the CUDA Deep Neural Network library (cuDNN) 8.6. All models trained in this study used 200 epochs. Batch sizes were 128 for CIFAR-10, CIFAR-100, Flowers-102, and STL-10, and 32 for Imagenette and Imagewoof. We used stochastic gradient descent with an initial learning rate of 0.1, momentum of 0.9, and weight decay of $5\times 10^{-4}$. The learning rate followed cosine annealing with linear warm-up during the first 5 epochs. Training images underwent random horizontal flipping and random cropping after padding, together with TrivialAugmentWide. Mixed precision, exponential moving averages, and early stopping were disabled to maintain comparable training conditions.

### 4.3. Evaluation Metrics and Statistical Methods

The primary metric is classification accuracy on the test set. The three-model comparison on CIFAR-100 also reports precision, recall, and F1 score. Each metric is computed separately for all 100 classes and then macro-averaged by taking its arithmetic mean across classes. To assess the stability of accuracy gains, the main comparisons and ablations on the four primary datasets were repeated independently using 3 random seeds (0, 1, and 2). We report means and standard deviations. All architectures use identical splits, augmentation procedures, and random seeds on a given dataset. Their prediction errors are therefore highly correlated, and the standard deviation of paired differences is smaller than the individual standard deviations. We use a one-sided *t*-test on seed-matched results for the directional hypothesis that GeoSieveNet outperforms ResNet-34, and report Cohen’s $d_{z}$ as the effect size. Significance conclusions across the 4 datasets use Holm correction.

### 4.4. Baseline Sources and Reproduction Protocol

The comparison results are divided into two groups. The first contains models reproduced in the same hardware and software environment: ResNet-34, ResNet-D, WideResNet-28-10, ConvNeXt-T, InternImage-T, DCNv4-S, HO-ResNet, and Multi-ResNet. These models use the same data splits, input dimensions, training epochs, optimizer settings, and augmentation pipeline as GeoSieveNet, allowing direct comparison. The second group contains results reported in the corresponding publications, including FAVOR+, ABNet-2G-R3, DAMSNet, SSCNet, MSMDFormer, ATONet, QKFormer, and some of the previously discussed Transformer baselines. Their backbones, input resolutions, and training procedures differ from our protocol. These values indicate the performance range of different approaches on the same datasets and are not used for a model-by-model ranking against GeoSieveNet. The table labels the groups as “Reproduced results” and “Literature-reported results,” and its caption specifies the input resolutions and training epochs for each group. InternImage-T represents modern DCNv3-based geometry-adaptive networks and provides a principal comparison with RDCM, which follows the same broad technical approach. Its original design targets 224×224 inputs and large-scale pretraining. At 32×32 resolution, its floating-point operation count is considerably higher than those of the other models; its accuracy and cost are compared only within the reproduced group. Multi-ResNet, an early residual-network improvement from 2016, is retained to show the gains achievable by stacking residual structures without changing sampling locations.

### 4.5. Network Configuration Selection

Predefined rotation-angle sets determine the direction groups in RDCM. Table 4 compares four configurations on CIFAR-100. Configuration 1 uses 0° and 90° and has the lowest parameter count and computational cost. Configuration 2 uses 0°, 45°, 90°, and 135°, whereas Configuration 3 uses 0°, 60°, 120°, and 180°. Configuration 4 uses eight directions from 0° to 157.5° at 22.5° intervals. Configuration 2 achieved the highest accuracy, 80.73%, and was used in the remaining RDCM configuration experiments. HGA placement was evaluated on CIFAR-100, Flowers-102, STL-10, and Imagenette. Flowers-102 and STL-10 were used for placement comparisons and the main comparisons, but not for module ablations. Table 3 summarizes the experimental role of each dataset.

The initial offset scale $s_{0}$ scales the predefined rotational displacement in Equation (1). With the angle set fixed to Configuration 2, we compared $s_{0}$ values from 0 to 1, as shown in Table 5. The value $s_{0}=0.1$ achieved the highest accuracy in this comparison on all four datasets and was adopted for RDCM.

**Figure 5:**
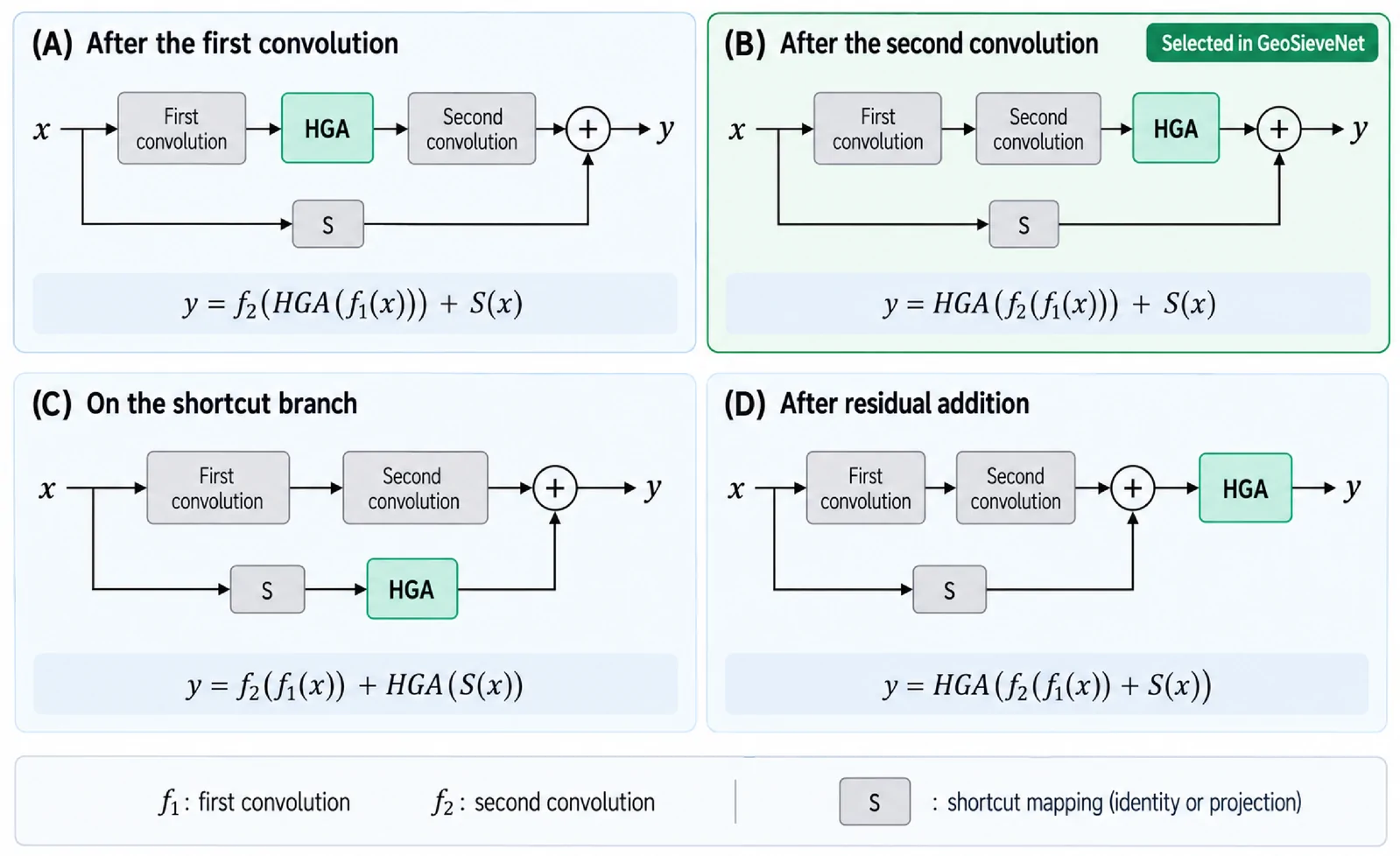
Candidate HGA insertion positions in a basic residual block. (**A**) After the first convolution. (**B**) After the second convolution and before residual addition, as adopted in this study. (**C**) After the shortcut mapping. (**D**) After residual addition. $f_{1}$ and $f_{2}$ denote the two convolutions, and *S* denotes an identity or projection shortcut mapping.

Figure 5 compares four HGA insertion positions. Let $f_{1}$ and $f_{2}$ denote the first and second convolutions, and let *S* denote the identity or projection shortcut mapping. Position A places HGA between the two convolutions; B places it after the second convolution and before residual addition. Position C places HGA after the shortcut mapping, and D places it after residual addition.

In Table 6, Position B achieved higher accuracy than the other candidates on all four datasets. HGA is therefore placed after the second convolution and before residual addition.

### 4.6. Classification Performance and Computational Cost

Table 7 reports the mean classification accuracies and standard deviations across 3 runs on CIFAR-10, CIFAR-100, Imagenette, and Imagewoof. GeoSieveNet achieved 96.95%, 81.86%, 94.71%, and 89.79%, respectively, exceeding ResNet-34 under the same training settings by 0.66, 3.06, 4.73, and 5.19 percentage points. The seed-wise paired differences were [+0.64,+0.64,+0.70] for CIFAR-10, [+3.01,+3.06,+3.12] for CIFAR-100, [+4.61,+4.66,+4.93] for Imagenette, and [+4.88,+5.30,+5.39] for Imagewoof. One-sided paired *t*-tests yielded p<0.001 on all four datasets, with Cohen’s $d_{z}$ values of 19.1, 55.6, 27.5, and 19.1, respectively. The largest gains occurred on Imagewoof and Imagenette, and the smallest on CIFAR-10, whose baseline accuracy of 96.29% already leaves less room for improvement.

On CIFAR-100, GeoSieveNet achieved higher precision, recall, and F1 score than ResNet-34 and WideResNet-28-10 in Table 8. The improvements over ResNet-34 were 3.60, 3.53, and 3.60 percentage points, respectively.

Table 9 reports parameter counts, FLOPs, and inference speed. Speed was measured on a single RTX 3090 using 32-bit floating point (FP32) and the channels_last memory format. Batch sizes were 128 for CIFAR datasets and 32 for Imagenette and Imagewoof, with the same input resolutions used for training. We performed 50 warm-up iterations followed by 200 timed iterations, with explicit synchronization every 50 iterations, and averaged 5 repetitions. Timing includes the forward pass and Softmax and top-*k* postprocessing, but excludes data loading and augmentation. GeoSieveNet has 25.65 M parameters, requires 5.73 G FLOPs, and has an inference latency of 4.12 ms, corresponding to 242.7 images/s. Relative to ResNet-34, parameters and FLOPs increase by 20.25% and 58.73%, respectively, while latency increases by 41.10%.

Table 10 relates computational cost to accuracy gains using relative changes. GeoSieveNet uses 1.20 times the parameters, 1.59 times the FLOPs, and 1.41 times the inference latency of the baseline. The absolute increases are 4.32 M parameters, 2.12 G FLOPs, and 1.20 ms. The corresponding relative accuracy increases are 0.69% on CIFAR-10, 3.88% on CIFAR-100, 5.26% on Imagenette, and 6.13% on Imagewoof. Cost multipliers are similar across the four datasets, whereas relative accuracy gains increase with the extent of geometric variation. The average cost per unit gain therefore decreases: the ratio of relative latency increase to relative accuracy increase is approximately 60 on CIFAR-10 and 10.6 on CIFAR-100, compared with 7.8 on Imagenette and 6.7 on Imagewoof. The same computational cost thus yields larger accuracy gains on datasets with substantial geometric variation. CIFAR-10 yields the smallest gain, so its deployment suitability depends on the available resources.

To determine whether the gains at these costs arise from the sampling mechanism itself, we constructed two controls under the same protocol (Table 11). The first, ResNet-34-W1.28, increases network width to reach 5.71 G FLOPs, closely matching the 5.73 G FLOPs of GeoSieveNet. At this budget, GeoSieveNet still improves accuracy by 0.54, 2.51, and 3.96 percentage points, showing that increased capacity alone does not explain the gains. The second, ResNet-34-W1.10, matches parameter count: its 25.81 M parameters are close to GeoSieveNet’s 25.65 M. Its accuracy remains 2.33–3.37 percentage points lower, while its FLOPs are also lower. Reducing the number of direction groups to 2 gives GeoSieveNet-Lite, which achieves accuracies of 96.72%, 80.32%, and 92.79% at 3.62 G FLOPs and 2.95 ms. At approximately the cost of ResNet-34, it retains accuracy gains of 0.43, 1.52, and 2.81 percentage points.

### 4.7. Ablation Results and Feature Visualization

Table 12 uses the complete RDCM as an ablation unit and includes an RDCM variant without FPM to isolate the contribution of FPM. All results are means and standard deviations over 3 runs; Table 13 reports significance tests. Adding HGA alone improved accuracy over ResNet-34 on all four datasets by 0.65–2.88 percentage points. Adding RDCM without FPM yielded gains of 0.25–3.19 percentage points. Enabling FPM within RDCM further improved accuracy by 0.36, 1.19, 1.42, and 1.89 percentage points, respectively. All gains were significant and increased with the extent of geometric variation. This indicates that preserving the fixed-grid branch constrains positional shifts introduced by deformable sampling. Adding HGA to the complete RDCM further increased accuracy by 0.05, 1.03, 0.13, and 1.14 percentage points, with larger complementary gains on CIFAR-100 and Imagewoof. The complete model achieved the highest accuracy on all four datasets.

**Figure 6:**
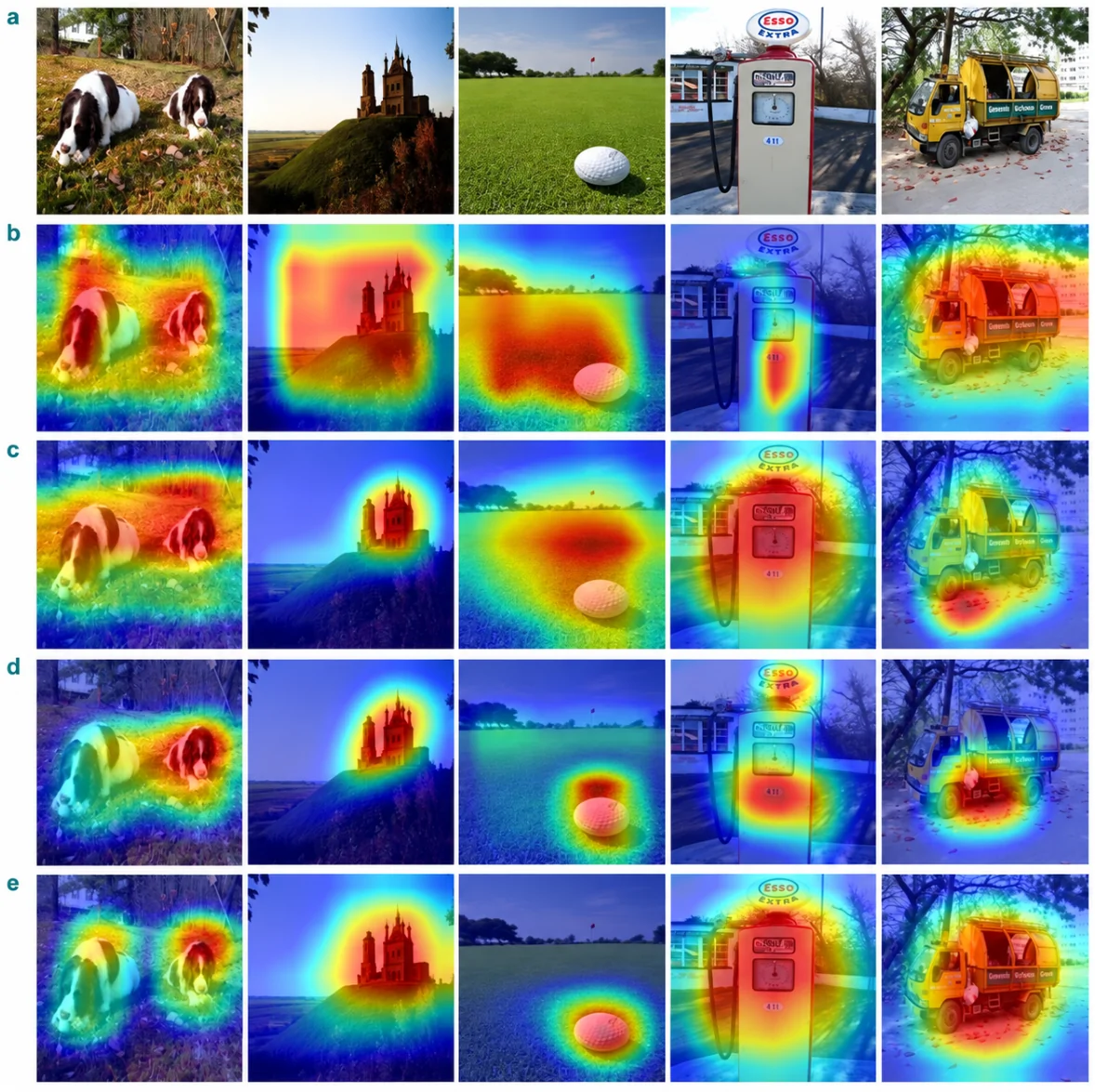
Activation distributions for Imagenette examples. (**a**) Original images. (**b**) DenseNet. (**c**) ResNet-34. (**d**) WideResNet-28-10. (**e**) GeoSieveNet. Warmer colors indicate stronger activation, whereas cooler colors indicate weaker activation.

Figure 6 compares activation distributions for examples from five Imagenette classes. We use gradient-weighted class activation mapping (Grad-CAM), targeting the output of the final residual stage of each backbone. Inputs are uniformly sized to 224×224, and the four models use the same weights and preprocessing across the visualization comparisons. DenseNet and ResNet-34 produce more dispersed high-response regions. WideResNet-28-10 produces more concentrated responses, although some remain in the background. GeoSieveNet responds mainly to the target object, with more concentrated responses on animal heads and building contours. Table 14 reports quantitative localization results for 200 Imagenette test images with bounding-box annotations. GeoSieveNet achieves a pointing game (PG) hit rate of 82.0%, exceeding ResNet-34 by 7.7 percentage points. The seed-wise differences are [+9.5,+5.5,+8.0], with a paired-test value of p=0.008. The intersection over union (IoU) between the class activation mapping (CAM) region and the target region, denoted CAM-IoU, is 0.512. The deletion area under the curve (AUC) decreases to 0.243. All three metrics are better than those of the compared models.

## 5. Discussion

### 5.1. Performance Changes and Architectural Contributions

RDCM produces larger gains than HGA when each is added independently on Imagenette and Imagewoof, making it the main source of improvement on these datasets. The independent FPM ablation shows that fusion of the fixed-grid and rotational deformable branches accounts for approximately half of RDCM’s gain. FPM contributes 1.42 and 1.89 percentage points on Imagenette and Imagewoof, respectively, compared with 0.36 percentage points on CIFAR-10. This difference is consistent with the datasets’ characteristics. Objects in Imagenette and Imagewoof show substantial pose and scale variation, making deformable sampling locations more likely to deviate from the regular grid. Retaining fixed-grid responses helps keep positional adjustments within an effective range. CIFAR-10 images have lower resolution and largely centered objects, leaving less spatial scope for sampling-location shifts.

Among the tested angle sets, the eight-direction configuration achieved lower accuracy than the four-direction Configuration 2. The initial offset-scale comparison likewise showed that increasing the predefined displacement did not yield sustained improvements. We therefore use four directions and $s_{0}=0.1$; Table 4 and Table 5 provide the detailed comparisons.

### 5.2. Accuracy Gains and Computational Overhead

GeoSieveNet’s accuracy gains accompany increases in parameters, computation, and inference latency under the same test conditions. Table 10 relates the return on this overhead to the extent of geometric variation. The ratio of relative latency increase to relative accuracy increase is approximately 60 on CIFAR-10, falling to 7.8 on Imagenette and 6.7 on Imagewoof. In the direct hardware comparison, GeoSieveNet achieves 94.71% accuracy on Imagenette at 4.12 ms. ResNet-34-W1.28, widened to match FLOPs, reaches 90.75% at 3.94 ms, leaving a 3.96-percentage-point accuracy gap at a similar latency scale. ResNet-34-W1.10 also achieves lower accuracy than GeoSieveNet despite its 25.81 M parameters. These controls attribute the gains to the sampling mechanism rather than capacity expansion; increasing width alone does not achieve the same performance.

For deployment, the number of direction groups provides a direct means of adjusting computational cost. Reducing this number to 2 gives GeoSieveNet-Lite, with 3.62 G FLOPs and 2.95 ms latency, close to ResNet-34’s 3.61 G and 2.92 ms. It still improves accuracy by 0.43, 1.52, and 2.81 percentage points on the three datasets. The available compute therefore determines the choice between the complete and lightweight configurations: four directions when resources permit, and two directions when resources are constrained or geometric variation is weaker.

## 6. Conclusions

We proposed GeoSieveNet, which combines dynamic sampling with predefined orientations and within-group feature weighting in ResNet-34. RDCM uses FPM to fuse fixed-grid and rotational deformable responses, while HGA generates gating weights from directional statistics and local responses. Across 3 independent runs on four primary classification datasets, GeoSieveNet achieved significantly higher accuracy than ResNet-34 under the same training settings. Gains on Imagenette and Imagewoof were 4.73 and 5.19 percentage points, respectively, with paired tests significant at the 0.001 level. Ablations attributed 0.36–1.89 percentage points to FPM alone, and HGA further improved accuracy when added to RDCM. At the same FLOPs budget as ResNet-34, GeoSieveNet retained an accuracy advantage of 0.54–3.96 percentage points. The lightweight configuration with 2 direction groups preserved an accuracy advantage at the baseline latency level, allowing computational cost to be adjusted to the available resources.

## Figures and Tables

**Table 1:** Implementation details of RDCM, FPM, and HGA. *K* is the kernel size, *D* is the number of direction groups, *N* is the number of channel groups, and $C_{g}=C/N$ is the number of channels per group. PWConv and DWConv denote pointwise convolution and depthwise convolution, respectively.

Module	Item	Value	Description
RDCM	Kernel size *K*	3	Regular 3×3 grid
RDCM	Direction groups *D*	4	0°, 45°, 90°, 135°
RDCM	Offset-prediction convolution	3×3, depthwise separable	Padding 1, stride 1
RDCM	Offset channels	$2DK^{2}=72$	Equally divided into 4 groups
RDCM	Offset-channel arrangement	18 channels per group	Paired as (Δx,Δy)
RDCM	Weight sharing across groups	No	Offset-prediction convolution shared
RDCM	Output spatial dimensions	H×W	16×16 at the input stage
RDCM	Interpolation	Bilinear, zero padding	Equations (6) and (7)
FPM	Weight branch	1×1, GN, GELU, 3×3, Sigmoid	Produces A
FPM	Preservation branch	3×3, BN, GELU	Produces P
FPM	Output	$F_{\mathrm{out}}=F⊙A+P$	Same dimensions as the input
HGA	Channel groups *N*	8	Independent gating in each group
HGA	Channels per group $C_{g}$	C/8	Determined by the stage channel count
HGA	Local branch	PWConv 1×1, DWConv 3×3	Produces H×W gating maps
HGA	Normalization	GN	Computed independently per group

**Table 2:** Datasets and splits used in this study. The training and test columns give the numbers of images actually used, and all compared models use the same test sets. Flowers-102 additionally contains 1020 official validation images, separate from the training and test sets.

Dataset	Input Size	Classes	Training	Test
CIFAR-10	32×32	10	50,000	10,000
CIFAR-100	32×32	100	50,000	10,000
Flowers-102	96×96	102	1020	6149
STL-10	96×96	10	5000	8000
Imagenette	224×224	10	9469	3925
Imagewoof	224×224	10	9025	3929

**Table 3:** Experiments performed on each dataset. A checkmark (✓) indicates inclusion, and a dash indicates non-inclusion. Configuration experiments cover the predefined rotation-angle sets and initial offset scale $s_{0}$; a dataset is marked if it is used in either experiment. Module ablations include the independent FPM ablation.

Experiment	CIFAR-10	CIFAR-100	Flowers-102	STL-10	Imagenette	Imagewoof
Configuration	✓	✓	–	–	✓	✓
HGA placement	–	✓	✓	✓	✓	–
Main comparisons	✓	✓	✓	✓	✓	✓
Module ablations	✓	✓	–	–	✓	✓
Cost and speed	✓	✓	–	–	✓	✓
Feature visualization	–	–	–	–	✓	–

**Table 4:** Comparison of rotation-direction configurations on CIFAR-100. FLOPs denotes floating-point operations.

Configuration	Accuracy/%	FLOPs/G	Parameters/M
1	79.88	1.432	25.462
2	**80.73**	1.463	25.630
3	80.48	1.463	25.630
4	80.69	1.534	25.971

Bold indicates the highest accuracy among the compared configurations.

**Table 5:** Effect of the initial offset scale $s_{0}$ on classification accuracy.

$s_{0}$	CIFAR-10/%	CIFAR-100/%	Imagenette/%	Imagewoof/%
0.0	96.49	80.73	94.34	88.75
0.1	**96.72**	**80.84**	**94.42**	**88.97**
0.2	96.66	80.12	94.26	88.75
0.3	96.66	79.86	94.34	88.69
0.4	96.60	80.17	94.39	88.62
0.5	96.46	80.58	94.36	88.87
0.6	95.53	80.79	94.26	88.67
0.7	96.55	80.50	94.34	88.34
0.8	96.52	80.43	94.31	88.69
0.9	96.49	80.43	93.88	88.26
1.0	96.56	80.39	94.29	87.96

Bold values indicate the highest accuracy in each accuracy column.

**Table 6:** Hierarchical gated attention (HGA) insertion-position experiments.

Position	CIFAR-100/%	Flowers-102/%	STL-10/%	Imagenette/%
A	79.74	90.35	80.37	92.20
B	**80.37**	**91.20**	**82.86**	**92.86**
C	79.88	86.44	75.62	91.03
D	80.09	88.76	79.78	91.54

Bold values indicate the highest accuracy in each accuracy column.

**Table 7:** Classification accuracy of different networks. The first group contains results reproduced in the same environment and with the same training settings as GeoSieveNet. Input sizes are 32×32 for CIFAR-10 and CIFAR-100, 96×96 for Flowers-102 and STL-10, and 224×224 for Imagenette and Imagewoof; all models are trained for 200 epochs. The second group contains literature-reported results obtained with different backbones, input resolutions, and training procedures. These values illustrate the performance range of different approaches and are not used for a model-by-model ranking against GeoSieveNet. A dash indicates that the cited source does not report the corresponding value.

Network	CIFAR-10	CIFAR-100	Flowers-102	STL-10	Imagenette	Imagewoof
*Reproduced results (same protocol as GeoSieveNet; means of 3 runs)*						
ResNet-34 [3]	96.29	78.80	89.41	78.62	89.98	84.60
ResNet-D [22]	96.35	79.24	90.12	79.48	90.21	84.92
WideResNet-28-10 [23]	95.87	80.50	91.06	80.15	88.34	78.71
ConvNeXt-T [24]	96.58	80.71	91.83	81.02	90.86	82.14
InternImage-T [13]	96.44	80.16	91.15	81.36	90.94	83.27
DCNv4-S [14]	96.52	80.38	91.62	81.71	91.27	83.84
HO-ResNet [25]	96.32	77.12	88.94	77.85	87.23	79.74
Multi-ResNet [26]	94.56	78.69	87.42	76.90	87.71	81.26
GeoSieveNet	**96.95**	**81.86**	**92.47**	**83.29**	**94.71**	**89.79**
*Literature-reported results (original training protocols)*						
FAVOR+ [27]	91.42	72.56	–	–	88.16	77.57
ABNet-2G-R3 [28]	96.08	80.83	–	–	–	–
DAMSNet [29]	96.51	80.50	–	–	–	–
SSCNet [30]	96.69	80.63	–	–	88.75	82.09
MSMDFormer [31]	95.65	77.46	–	–	–	–
ATONet [32]	94.51	78.56	–	–	86.68	80.21
QKFormer [33]	96.18	80.27	–	–	88.42	81.67

Bold values indicate the highest accuracy in each dataset column within the reproduced group.

**Table 8:** Precision, recall, and F1 score on CIFAR-100. All three metrics are computed per class and then macro-averaged.

Network	Precision/%	Recall/%	F1/%
ResNet-34	78.31	78.28	78.20
WideResNet-28-10	80.51	80.42	80.39
GeoSieveNet	**81.91**	**81.81**	**81.80**

Bold values indicate the highest value in each metric column.

**Table 9:** Parameter counts, FLOPs, inference throughput, and latency. Throughput and latency are measured under the same conditions. Timing includes Softmax and top-*k* postprocessing and excludes data loading and augmentation.

Network	Parameters/M	FLOPs/G	Throughput/ (Images·$s^{-1}$)	Latency/ms
ResNet-34 [3]	21.33	3.61	342.5	2.92
ResNet-D [22]	21.85	3.68	336.1	2.98
WideResNet-28-10 [23]	36.50	5.96	216.4	4.62
ConvNeXt-T [24]	28.59	4.46	268.9	3.72
InternImage-T [13]	29.87	22.53	128.4	7.79
DCNv4-S [14]	31.20	18.96	151.7	6.59
HO-ResNet [25]	50.26	11.05	178.2	5.61
Multi-ResNet [26]	51.23	11.73	160.3	6.24
GeoSieveNet	25.65	5.73	242.7	4.12

**Table 10:** Computational cost and accuracy gains of GeoSieveNet relative to ResNet-34. A relative increase is the percentage change from the baseline value. The relative cost ratio divides the relative latency increase by the relative accuracy increase. Absolute accuracy gains are expressed in percentage points (pp).

Dataset	Relative Latency Increase/%	Relative Accuracy Increase/%	Relative Cost Ratio	Absolute Accuracy Gain/pp
CIFAR-10	41.10	0.69	59.96	0.66
CIFAR-100	41.10	3.88	10.57	3.06
Imagenette	41.10	5.26	7.81	4.73
Imagewoof	41.10	6.13	6.70	5.19

**Table 11:** Comparisons at matched FLOPs and parameter budgets. ResNet-34-W1.10 and ResNet-34-W1.28 use width multipliers of 1.10 and 1.28, respectively. The former matches GeoSieveNet’s parameter count, and the latter matches its FLOPs. GeoSieveNet-Lite reduces the number of direction groups to D=2, bringing its FLOPs close to those of ResNet-34. All models use the same training protocol.

Network	Parameters/M	FLOPs/G	Latency/ms	CIFAR-10	CIFAR-100	Imagenette
*Baseline*						
ResNet-34	21.33	3.61	2.92	96.29	78.80	89.98
*Capacity and cost controls (width or direction groups adjusted to the budget)*						
ResNet-34-W1.10	25.81	4.25	3.42	96.34	79.21	90.42
ResNet-34-W1.28	31.42	5.71	3.94	96.41	79.35	90.75
GeoSieveNet-Lite (D=2)	24.10	3.62	2.95	96.72	80.32	92.79
*Complete proposed model*						
GeoSieveNet	25.65	5.73	4.12	**96.95**	**81.86**	**94.71**

Bold values indicate the highest accuracy in each accuracy column.

**Table 12:** Ablation experiments for the rotational deformable convolution module (RDCM), feature preservation module (FPM), and hierarchical gated attention (HGA). Values are means ± standard deviations over 3 random seeds, with each model trained 3 times on each dataset.

Network	CIFAR-10/%	CIFAR-100/%	Imagenette/%	Imagewoof/%
ResNet-34	96.29 ± 0.06	78.80 ± 0.08	89.98 ± 0.11	84.60 ± 0.18
+HGA	96.85 ± 0.17	80.40 ± 0.06	92.86 ± 0.08	85.25 ± 0.25
+RDCM (without FPM)	96.54 ± 0.06	79.64 ± 0.05	93.17 ± 0.07	86.76 ± 0.02
+RDCM (with FPM)	96.90 ± 0.06	80.83 ± 0.08	94.58 ± 0.06	88.65 ± 0.07
+HGA+RDCM (without FPM)	96.90 ± 0.15	80.79 ± 0.25	94.32 ± 0.11	89.02 ± 0.36
GeoSieveNet	**96.95 ± 0.08**	**81.86 ± 0.13**	**94.71 ± 0.16**	**89.79 ± 0.19**

Bold values indicate the highest mean accuracy in each dataset column.

**Table 13:** Significance tests for the main accuracy gains. One-sided paired *t*-tests use results matched by random seed, with 2 degrees of freedom. $s_{d}$ is the sample standard deviation of paired differences, and *p* is the one-sided value before Holm correction. Statistics are calculated from seed-wise results and then rounded consistently. FPM rows compare RDCM with FPM against RDCM without FPM.

Comparison	Dataset	Gain/pp	$s_{d}$	t(2)	*p*
GeoSieveNet − ResNet-34	CIFAR-10	+0.66	0.035	33.0	<0.001
	CIFAR-100	+3.06	0.055	96.3	<0.001
	Imagenette	+4.73	0.172	47.6	<0.001
	Imagewoof	+5.19	0.272	33.0	<0.001
FPM (with − without)	CIFAR-10	+0.36	0.017	36.0	<0.001
	CIFAR-100	+1.19	0.050	41.2	<0.001
	Imagenette	+1.42	0.107	22.9	<0.001
	Imagewoof	+1.89	0.085	38.3	<0.001

**Table 14:** Quantitative localization results for activation maps. Grad-CAM is evaluated on 200 Imagenette test images with bounding boxes, targeting the output of each backbone’s final residual stage. PG denotes pointing game, and PG-E denotes energy-based pointing game; both are reported as means and standard deviations over 3 random seeds. Deletion-AUC and insertion-AUC are means over the same test images. Lower deletion-AUC and higher insertion-AUC indicate more effective deletion and insertion, respectively.

Network	PG/%	PG-E/%	CAM-IoU	Deletion-AUC	Insertion-AUC
DenseNet	66.50 ± 1.32	54.2 ± 2.1	0.361	0.312	0.684
ResNet-34	74.33 ± 1.61	61.5 ± 1.8	0.418	0.284	0.731
WideResNet-28-10	78.17 ± 0.76	66.1 ± 1.7	0.463	0.261	0.775
GeoSieveNet	**82.00 ± 1.00**	**71.4 ± 1.3**	**0.512**	**0.243**	**0.812**

Bold values indicate the best result in each metric column.

## Data Availability

CIFAR-10 and CIFAR-100 are available from https://cave.cs.toronto.edu/kriz/cifar.html (University of Toronto dataset page) (accessed on 8 October 2026). Flowers-102 is available from https://www.robots.ox.ac.uk/~vgg/data/flowers/102/ (Oxford VGG dataset page) (accessed on 8 October 2026) and STL-10 from https://cs.stanford.edu/~acoates/stl10/ (Stanford dataset page) (accessed on 8 October 2026). Imagenette and Imagewoof are available from https://github.com/fastai/imagenette (fastai project repository) (accessed on 8 October 2026). Table 2 specifies the splits used in this study. Summarized experimental results are presented in the main text, figures, and tables.
